# Promoting resilience of large international collaborative research programs in times of global crisis

**DOI:** 10.1002/ece3.6835

**Published:** 2020-09-27

**Authors:** Stefan Trogisch, Georg Albert, Jianqing Du, Yanfen Wang, Kai Xue, Helge Bruelheide

**Affiliations:** ^1^ Institute of Biology/Geobotany and Botanical Garden Martin Luther University Halle‐Wittenberg Halle (Saale) Germany; ^2^ German Centre for Integrative Biodiversity Research (iDiv) Halle‐Jena‐Leipzig Leipzig Germany; ^3^ Institute of Biodiversity Friedrich Schiller University Jena Jena Germany; ^4^ University of Chinese Academy of Sciences Beijing China; ^5^ Yanshan Mountains Earth Critical Zone and Surface Flux Research Station Chinese Academy of Sciences Beijing China; ^6^ CAS Center for Excellence in Tibetan Plateau Earth Sciences Beijing China

**Keywords:** BEF‐China, biodiversity, coronavirus, COVID‐19, early‐career researchers, TreeDì

## Abstract

The recent coronavirus disease (COVID‐19) is impacting the research community worldwide with unforeseen long‐term consequences for research, doctoral training, and international collaboration. It is already clear that the immediate effects of the crisis resulting from disrupted research stays and reduced career development opportunities are being most detrimental to early‐career researchers. Based on a Sino‐German international research training group dedicated to doctoral training and biodiversity‐ecosystem functioning research, we show how resilience of large collaborative research programs can be promoted in times of global crisis. We outline possible adaptations in the areas of funding, research, teaching and learning, supervision and mentoring, and international collaboration helping to reduce detrimental impact for early‐career researchers and to permanently strengthen the performance of large collaborative research groups in the postpandemic era.

## Graphical abstract

The recent coronavirus disease is impacting the research community worldwide with unforeseen long‐term consequences for research, doctoral training, and international collaboration. Taking a joint international research training group between China and Germany as an example, we illustrate how the resilience of large international collaborative research programs can be promoted in times of global crisis.

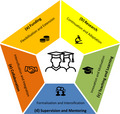

## 1 INTRODUCTION

The recent COVID‐19 pandemic has undoubtedly impacted the research community worldwide and has triggered remarkable shifts in how we are doing research, teach, and learn (Corlett et al., 2020). While the long‐lasting impacts on the research and education landscape are yet difficult to predict, the immediate effects could not be more apparent. Disrupted fieldwork and research stays abroad, cancelled conferences and meetings and a complete suspension of classroom seminars and courses have brought traditional academic life, as we know it, to a sudden halt.

Among the academic groups, early‐career researchers and in particular PhD students may be most affected by the current situation: Data collection is virtually impossible in many regions of the world due to travel restrictions and temporary shutdown of field stations, and network and career development opportunities are fundamentally reduced (Paula, 2020). While prioritizing analyses of already existing data and manuscript preparation can partly buffer negative consequences, there is uncertainty how the overall quality of PhD theses and papers, time to degree and job prospects are affected by the pandemic. Universities, nonuniversity research institutions, funding agencies and policymakers have now the obligation to minimize the detrimental impact for early‐career researchers. Here, we show how this can be achieved based on the example of an international research training group with doctoral researchers spatially located both in Germany and China. Based on our experience, we outline which measures can be taken and extract basic principles of how the resilience of large international research programs can be promoted in times of global crisis.

## 2 INTERNATIONAL RESEARCH TRAINING GROUPS IN THE RECENT COVID‐19 PANDEMIC

A high degree of international collaboration and mutual research visits are key elements of international research training groups that are jointly operated by a research institution or consortium in one country, in our case Germany, and an equivalent partner institution abroad (DFG, 2010). As a key mission, international research training groups aim at promoting international research networks and successful careers for their doctoral researchers. Usually, these structured programs involve between 10 and 15 doctoral researchers from both partner countries, respectively. All doctoral researchers are working on specific projects that are linked by a common research theme and benefit from a joint qualification program and reciprocal research visits.

**Figure 1:**
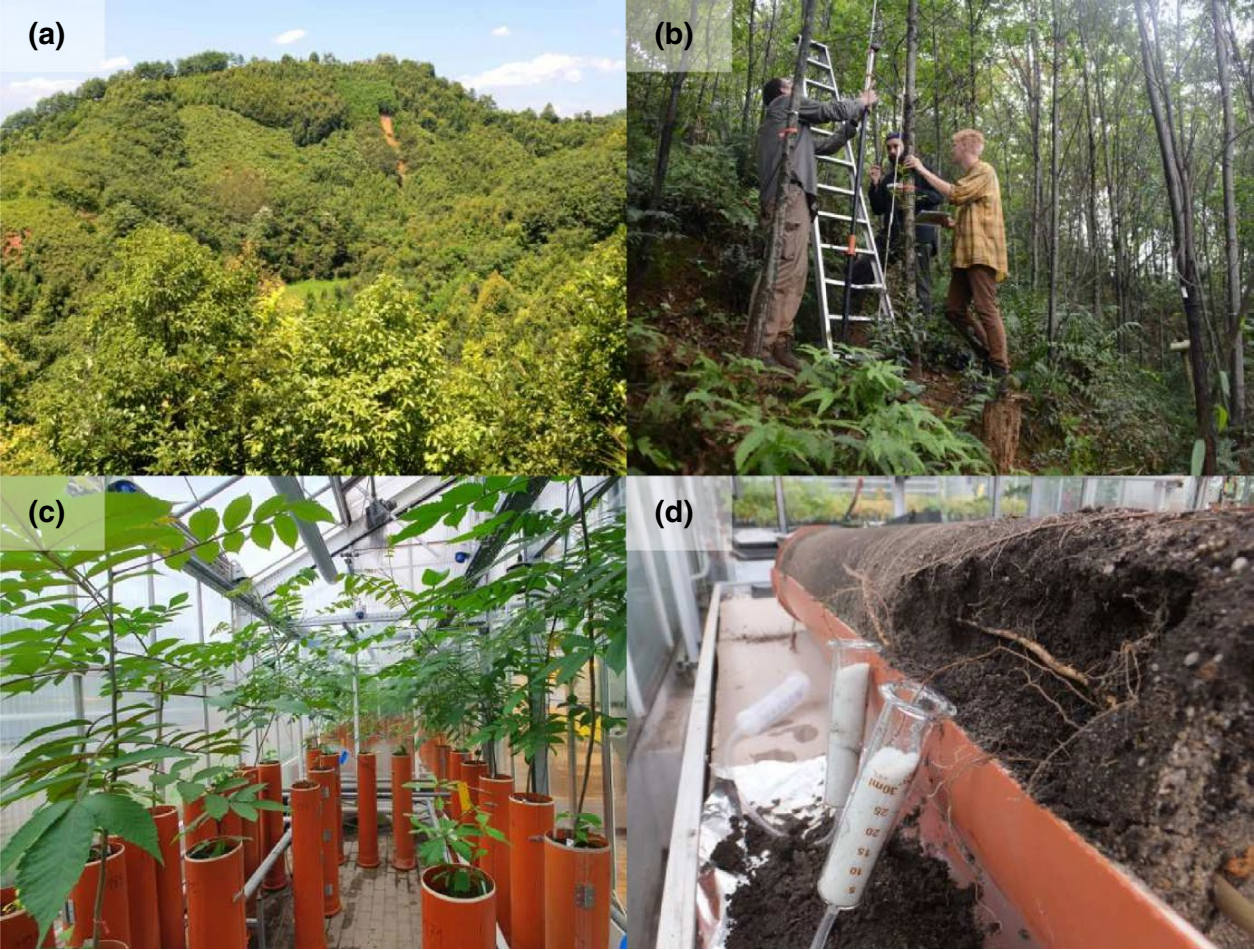
The current pandemic has impacted ongoing fieldwork of many early‐career researchers in the field of ecology and evolution due to international travel restrictions and other containment measures. Limited access to remote field sites, such as the tree diversity experiment "BEF‐China" (a), prevents data collection (b). Fallback plans involving, for example, local greenhouse studies (c) are a good strategy to acquire original research data needed for the completion of PhD theses. Such experiments do not only allow to study additional topics (e.g., root exudation patterns (d)) and the testing of new sampling methods but also offer added value in undergraduate training

In our example, the International Research Training Group "TreeDì" (www.treedi.de), doctoral researchers study the role of tree‐tree interactions and local tree diversity for ecosystem functioning in a large tree diversity experiment ("BEF‐China," Figure 1a,b) located in East China (Bruelheide et al., 2014). Courses and workshops are alternately organized at the German Centre for Integrative Biodiversity Research (iDiv) in Germany and the University of Chinese Academy of Sciences (UCAS) in Beijing. Usually, doctoral researchers of our structured program spend a total of 6–12 months in the field with an additional 6‐month stay in their reciprocal partner research groups in Germany or China. With field stations and laboratory facilities having been closed and strict travel restrictions in place, severe consequences for individual PhD projects are often unavoidable, in particular, if repeated or seasonal measurements cannot be continued. Furthermore, the half‐year research stays of PhD students from China in Germany, originally planned in 2020, as well as reciprocal visits in China had to be cancelled. As a consequence, the face‐to‐face qualification program of 2020 including for example excursions, retreats, and an on‐site conference became obsolete and required a thoroughly replanning and adaptation to the new situation.

Many large collaborative research consortia and individual PhD projects in the field of ecology and evolution are currently facing similar challenges (Inouye et al., 2020). That the recent pandemic may be most detrimental to early‐career researchers relying on data collection at field sites or laboratories abroad is of particular concern. Funding agencies worldwide have responded to the situation by adapting their regulations allowing, for example, the transfer of money to 2021 or application for extra funds to compensate for pandemic‐related delays in ongoing research projects (Stoye, 2020, Figure 2a). For instance, the German Research Foundation (DFG) has decided to fund up to three additional monthly payments for doctoral researchers of research training groups if already approved funds are not sufficient to cover extra costs caused by the current crisis. However, with an impending global depression unfolding, increasing national debts and budget cuts, long‐term prospects of large collaborative research programs are more than uncertain.

**Figure 2:**
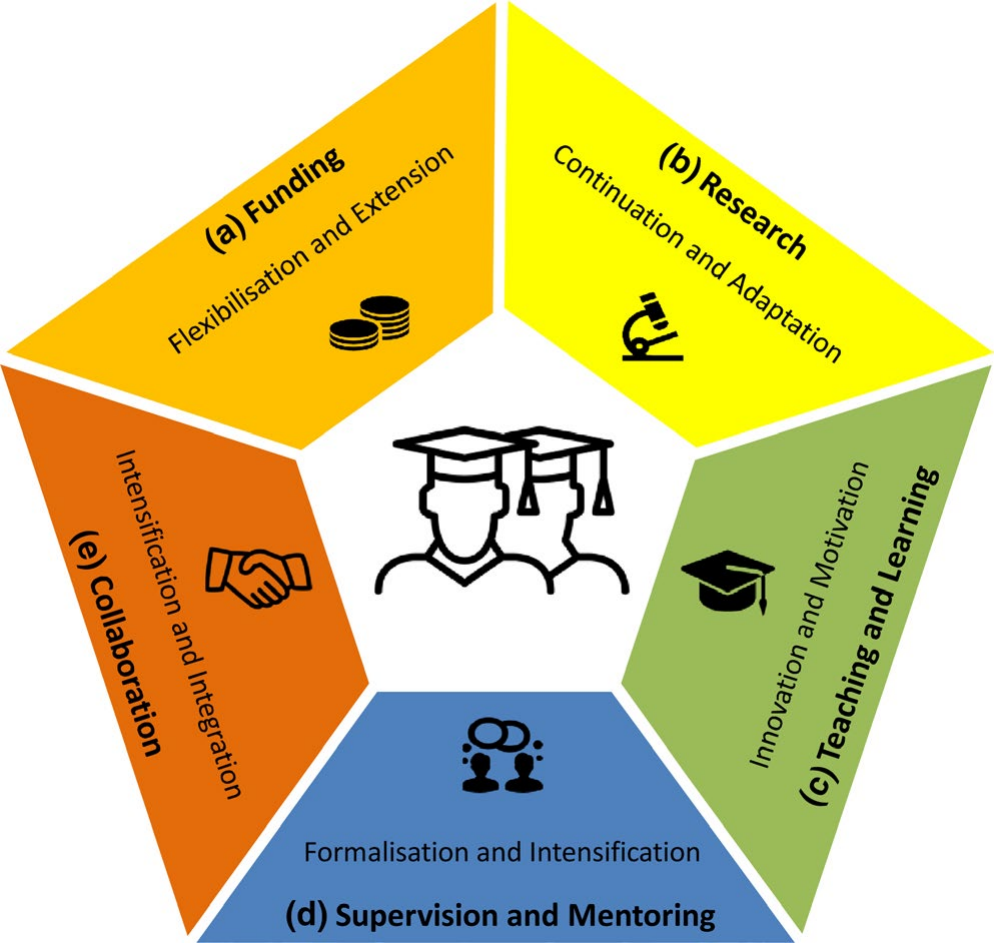
The resilience of large international collaborative research programs against global crises depends on five pillars. First, funding and reporting deadlines have to become more flexible and should be extended (a). Second, the continuation of research (b) should be promoted by adaptation and implementation of fallback plans (e.g., meta‐analyses, laboratory and greenhouse experiments in case fieldwork abroad is not possible). Third, graduate qualification programs require innovation in teaching (online courses, distance learning, innovative communication tools) promoting also the learning motivation of early‐career researchers (c). Fourth, supervision and mentoring (d) require formalization and intensification to ensure continuous support and help in case of mental health problems. As fifth pillar, international collaboration (e) needs to be intensified and should become more integrative (e.g., by regular online meetings and virtual seminars) to foster mutual trust which is the basis for good communication as well as for creative and successful teamwork

## 3 PROMOTING RESILIENCE IN RESEARCH

The most severe implication resulting from the current epidemic may be the restricted access to field sites to collect data. As before, the work plan of each PhD project should therefore contain a statement on risks and outline possible strategies to avoid or cope with them. The recent crisis has impressively reiterated the importance of thorough risk analyses and fallback plans doctoral researchers and supervisors should agree on at the start of the PhD project. We recommend that large collaborative research initiatives dedicated to doctoral training feature overarching strategies on how to reduce risks and uncertainties for involved doctoral researchers (Figure 2b).

In our case, we have implemented two general fallback instruments. Firstly, a greenhouse experiment (Figure 1c,d) using the same species as planted in China but set up in Germany to study tree interactions under various treatments (tree species richness, fertilization, soil inoculum). The complementary experiment allows approaches that are not easily possible in the field, such as destructive harvests, working under (semi‐)sterile conditions, application of stable isotopes and manipulating abiotic (e.g., soil moisture) and biotic conditions (e.g., soil inoculation). In this way, early‐career researchers have the opportunity to learn the principles of experimental designs to set up and analyze their own experiment. In addition, the greenhouse experiment is an excellent opportunity to involve and train undergraduate students who would have done fieldwork at the sites in China. Secondly, all doctoral researchers are encouraged to work on a synthesis paper by making use of data that have already been collected since the establishment of the BEF‐China tree diversity experiment in 2009. A well‐maintained database following FAIR data principles (Wilkinson et al., 2016) is a prerequisite to locate and remobilize available project data (Nadrowski et al., 2013). Besides performing a synthesis study or meta‐analysis, the collaboration with and integration within larger research networks, in our case the global network of tree diversity experiments "TreeDivNet" (Paquette et al., 2018), is a good way to further reduce the direct dependency on field data collection during the recent pandemic while still being able to author high‐quality papers.

## 4 PROMOTING RESILIENCE IN TEACHING AND LEARNING

Triggered by the global crisis, academia has experienced an unprecedented boost in online teaching and learning. For example, more than 4,400 undergraduate and graduate courses had to be offered online at Peking University to fulfill national teaching obligations (Bao, 2020). Based on the experience collected in China and worldwide, important guidelines have been extracted to make online education successful for both teachers and students (Bao, 2020; Gewin, 2020). One key aspect is to stimulate active learning of students by alternating teaching phases, ideally divided into short modules of 20 min, and offline self‐learning phases (Bao, 2020). However, the effort for designing suitable online courses specifically tailored to the needs of early‐career researchers should not be underestimated. To maximize course offers for doctoral researchers of the own program, we advise to cooperate with larger graduate academies at universities and to negotiate course access even for doctoral researchers not registered at the respective university but belonging to the research training group. The number of high‐quality online courses, workshops, and talks offered by the universities has seen an unprecedented boost since the advent of the crisis. For our doctoral researchers, whether based in Germany or China, this means that they can attend additional online courses offered by the universities of Halle, Jena and Leipzig, the Graduate School yDiv of the German Centre for Integrative Biodiversity Research (iDiv) as well by the cooperating institutions in China like the University of Chinese Academy of Sciences.

While synchronous formats, for example, live online seminars, scheduled at specific times allow students to get instant feedback and questions answered, we also clearly advocate making use of and developing innovative program‐specific asynchronous learning formats (Figure 2c). Realized in learning management systems they allow students a self‐paced learning experience. By combining different formats (e.g., video, audio, text, and figures) and interactive components (e.g., quizzes, chats, etherpads), such e‐learning modules can provide true benefits and promote students' self‐motivation.

Although a wide range of commercial and free online courses are available (MOOCs—Massive Open Online Courses; for example, www.mooc.org, www.coursera.org) (Kaplan & Haenlein, 2016), they are often too general and not tailored to early‐career researchers specialized in the field of ecology and evolution. For example, available courses on scientific writing or statistics designed for other fields, such as physics, may allow to acquire important knowledge but this has to be laboriously transferred to one's own scientific field. We therefore encourage the development of own asynchronous online courses (SPOCs—Small Private Online Courses) (Kaplan & Haenlein, 2016) to create synergies and added values for early‐career researchers enrolled in a specific doctoral program. For instance, an online course on scientific writing and publishing can explain the general principles based on papers written by members of the own research program. Similar, how to review scientific papers can be showcased as group peer‐review on students' own paper drafts. Etherpads with track changes and comment mode are suitable tools and help early‐career researchers to collect feedback on their drafts from members of the research program. As one major advantage, the content of e‐learning modules can be gradually extended and improved at relatively low effort for new doctoral researchers joining the program. Another e‐learning module we are currently preparing is an introduction to the experimental design of the tree diversity experiment "BEF‐China." Given the complexity of the experiment with more than 500 plots established at two sites, high number of different tree species pools and extinction scenarios, we think that this could be a great help for any new researcher either planning to work with already collected data or considering fieldwork in the experiment. An integrated quiz for matching tree species names with pictures underpinned with species information could easily convey taxonomic knowledge before the start of fieldwork.

## 5 PROMOTING RESILIENCE IN SUPERVISION AND MENTORING

Supervision and mentoring of early‐career researchers have increased in importance given the challenges posed by the global health crisis. Besides difficulties in keeping up with ambitious research plans and arising funding insecurities, early‐career researchers are facing additional problems if they cannot travel to their home country or place of work (Ma & Miller, 2020). The resulting high degree of uncertainty increases the risk of mental health problems, particularly among early‐career researchers (Levecque et al., 2017). Supervisors and mentors take on a special role in providing advice on mental well‐being and where to seek professional help in case of problems. Based on our experience, a PhD advisory committee composed of the first and second supervisor and additional members with complementary scientific background and expertise may be a beneficiary solution to provide guidance and support for doctoral researchers. During challenging times, shorter but more frequent meetings with the committee or single members allow the timely adaptation to rapidly changing external factors in order to reduce uncertainties for doctoral researchers (Figure 2d). A more formalized and structured supervision and mentoring system including a supervision agreement, a detailed work plan, and regular meetings with the PhD advisory committee to discuss progress reports can help to better manage unforeseen challenges (Figure 2d). In addition, a PhD advisory committee is essential to ensure reliable supervision and guidance in the case that single committee members are being affected by the pandemic in their professional and personal lives and becoming temporarily unavailable.

## 6 INTERNATIONAL COLLABORATION IN THE POSTPANDEMIC PERIOD

The digital revolution currently taking place at the majority of academic institutions will influence how we teach and learn, but also how we collaborate in the future. Remarkably, the recent pandemic has prompted an infrastructural capacity building in online teaching and learning unconceivable at the beginning of 2020. With a wide range of online tools at hand and the habituation of the global scientific community to online conferences, meetings, and lectures, academic life, as we know it, could be increasingly transferred into the digital space.

While this could yield many positive aspects, for example, combining team members based on expertise without geospatial bias but also saving of time, travel costs, and CO_2_ emissions (Rudolph et al., 2020), there might be also serious drawbacks arising if this trend continues. For example, international collaboration could remain digital also in the postpandemic period if the digital infrastructure were taken as an excuse by funding agencies to cut travel funds. However, we believe that personal contacts are indispensable to initiate new and maintain collaborations based on mutual trust which is the basis for good communication as well as for creative and successful teamwork. This may be especially true for culturally heterogenous teams. Therefore, we recommend to meet face to face whenever this is possible and necessary and to additionally make use of available online communication tools to intensify collaboration and joint supervision. This will not only intensify cross‐border collaboration but will also ensure integration of all team members which is particularly important for early‐career researchers (Figure 2e).

## 7 CONCLUSIONS

Taking a joint international research training group between China and Germany as an example, we have illustrated the challenges the current pandemic poses for large collaborative and multi‐national research programs. Based on our experience, the uncertainties for early‐career researchers can be reduced by implementing a number of measures in the fields of funding, research, teaching and learning, supervision and mentoring, and collaboration. Although the COVID‐19 crisis has disrupted research plans, the forced change in perspective has unveiled new opportunities, for example, in distance learning and remote collaboration. By extracting and implementing those adaptations that promise positive effects also in the post‐pandemic period, the resilience and performance of large collaborative research programs could be permanently strengthened. This is not only of utmost importance for the education and training of early‐career researchers in an international context but also for advancing ecological research by fostering knowledge transfer and exchange. As impressively shown by the recent pandemic, effective international collaboration will eventually be key to tackle global environmental problems now and in the future.

## CONFLICT OF INTEREST

None declared.

## AUTHOR CONTRIBUTION

**Stefan Trogisch:** Conceptualization (lead); Project administration (lead); Resources (equal); Visualization (lead); Writing‐original draft (lead); Writing‐review & editing (lead). **Georg Albert:** Conceptualization (equal); Visualization (equal); Writing‐original draft (equal); Writing‐review & editing (equal). **Jianqing Du:** Conceptualization (equal); Project administration (equal); Visualization (equal); Writing‐original draft (equal); Writing‐review & editing (equal). **Yanfen Wang:** Conceptualization (equal); Funding acquisition (lead); Project administration (lead); Resources (lead); Visualization (equal); Writing‐original draft (equal); Writing‐review & editing (equal). **Kai Xue:** Conceptualization (equal); Funding acquisition (equal); Project administration (equal); Visualization (equal); Writing‐original draft (equal); Writing‐review & editing (equal). **Helge Bruelheide:** Conceptualization (equal); Funding acquisition (lead); Project administration (lead); Resources (lead); Supervision (lead); Visualization (equal); Writing‐original draft (equal); Writing‐review & editing (equal).

## Data Availability

There are no data associated with this article.
